# Long-term survival, toxicities, and the role of chrono-chemotherapy with different infusion rates in locally advanced nasopharyngeal carcinoma patients treated with intensity-modulated radiation therapy: a retrospective study with a 5-year follow-up

**DOI:** 10.3389/fonc.2024.1371878

**Published:** 2024-03-22

**Authors:** Lina Liu, Xunyan Luo, Weili Wu, Yuanyuan Li, Jinhua Long, Xiuling Luo, Xiaoxiao Chen, Xiuyun Gong, Chaofen Zhao, Qianyong He, Zhuoling Li, Kai Shang, Yue Chen, Xu Xinyu, Feng Jin

**Affiliations:** ^1^ Department of Oncology, Affiliated Hospital of Guizhou Medical University, Guiyang, Guizhou, China; ^2^ School of Clinical Medicine, Guizhou Medical University, Guiyang, Guizhou, China; ^3^ Department of Oncology, The Affiliated Cancer Hospital of Guizhou Medical University, Guiyang, Guizhou, China

**Keywords:** nasopharyngeal carcinoma, chrono-chemotherapy, radiotherapy, intensity-modulated radiotherapy, late toxicity

## Abstract

**Purpose:**

This study aimed to evaluate 5-year outcomes and the late toxicity profile of chrono-chemotherapy with different infusion rates in patients with locally advanced nasopharyngeal carcinoma (NPC).

**Methods and materials:**

Our retrospective analysis included 70 patients with locally advanced NPC stages III and IVB (according to the 2010 American Joint Committee on Cancer staging system). Patients were treated with two cycles of induction chemotherapy (IC) before concurrent chemoradiotherapy (CCRT) at Guizhou Cancer Hospital. The IC with docetaxel, cisplatin (DDP) and fluorouracil regimen. Patients were divided into two groups during CCRT. Using a “MELODIE” multi-channel programmed pump, DDP (100 mg/m^2^) was administered for 12 hours from 10:00 am to 10:00 pm and repeated every 3 weeks for 2-3 cycles. DDP was administered at the peak period of 4:00 pm in the sinusoidal chrono-modulated infusion group (Arm A, n=35). The patients in Arm B received a constant rate of infusion. Both arms received radiotherapy through the same technique and dose fraction. The long-term survival and disease progression were observed.

**Results:**

After a median follow-up of 82.8 months, the 5-year progression-free survival rate was 81.3% in Arm A and 79.6% in Arm B (P = 0.85). The 5-year overall survival rate was not significantly different between Arm A and Arm B (79.6% vs 85.3%, P = 0.79). The 5-year distant metastasis-free survival rate was 83.6% in Arm A and 84.6% in Arm B (P = 0.75). The 5-year local recurrence-free survival rate was 88.2% in Arm A and 85.3% in Arm B (P = 0.16). There were no late toxicities of grade 3-4 in either group. Both groups had grade 1-2 late toxicities. Dry mouth was the most common late toxic side effect, followed by hearing loss and difficulty in swallowing. There was no statistically significant difference between Arm A and Arm B in terms of side effects.

**Conclusion:**

Long-term analysis confirmed that in CCRT, cisplatin administration with sinusoidal chrono-modulated infusion was not superior to the constant infusion rate in terms of long-term toxicity and prognosis.

## Introduction

Nasopharyngeal carcinoma (NPC) is considered a rare form of cancer globally. However, high incidence in specific geographic and ethnic populations is noteworthy (1, 2). NPC is endemic in Southeast Asia and Southern China, particularly in the Guangdong province (3). Platinum-based concurrent chemoradiotherapy (CCRT) combined with induction chemotherapy (IC) or adjuvant chemotherapy (AC) is the standard treatment regimen for locally advanced NPC. However, studies have shown that compared to CCRT-AC strategy, IC-CCRT offers the advantages of better tolerance and early eradication of micro metastases (4, 5). CCRT has been shown to have the highest benefit in patients with locally advanced NPC. The specific regimen includes commonly used DDP at 100 mg/m^2^ every 3 weeks during intensity modulated radiation therapy (IMRT) for 2-3 cycles. However, adding DDP chemotherapy to radiotherapy increases the incidence of treatment-related toxic side effects, which reduces patient treatment compliance and quality of life (6–8).

Chrono-chemotherapy is based on the changes of the biological rhythm. If administered at appropriate times, not only can it reduce the adverse reactions of chemotherapy and improve the quality of life, it can also improve the immune function (9–12). Studies have shown that using a “MELODIE” multi-channel programmed pump during sinusoidal chrono-modulated infusion for IC in NPC, the DDP infusion time lasts from 10:00am to 10:00pm, and the peak delivery time occurs at 4:00 pm. The patients in the second group received infusions at a constant rate. As a result, chrono-chemotherapy significantly reduced stomatitis but did not show a superior therapeutic response (11, 13).

In our previous study, we compared the advantages and disadvantages of DDP administered through sinusoidal chrono-modulated infusion and at a constant rate of infusion to investigate the role of chrono-chemotherapy in CCRT for NPC. We found no significant difference in acute toxic side effects; efficacy; and the 2-year overall survival (OS), progression-free survival (PFS), and disease-free survival (DFS) between the two groups. However, the sinusoidal chrono-modulate infusion group showed improved T cell immunity (14). Herein, we aimed to report the updated 5-year detailed analyses of survival outcomes and late toxic effects to assess the ultimate therapeutic efficacy of sinusoidal chrono-modulated infusion and the constant infusion rate during CCRT.

## Materials and methods

### Patient selection

We included 70 patients with locally advanced non-keratinizing NPC (type II/III World Health Organization classification) who were treated in Guizhou Cancer Hospital between December 2013 and March 2017. NPC was newly diagnosed and confirmed by biopsy. Follow-up data were evaluated retrospectively. According to the different infusion rates of cisplatin during CCRT, the patients were divided into sinusoidal chrono-modulated infusion group (Arm A) and constant rate infusion group (Arm B).

The eligibility criteria were (1) newly diagnosed pathology stage III, IVa, and IVb NPC by (according to the 2010 American Joint Committee on Cancer [AJCC] staging system) and receiving initial treatment; (2) between 18 and 70 years old;(3) normal hematologic, kidney, and liver function; (4) the Karnofsky Performance Status (KPS) Scale score of 70 or higher and (5) no distant metastasis. The exclusion criteria were (1) contraindications to radiotherapy or chemotherapy; (2) previous treatment for NPC;(3) prior or synchronous malignant disease; (4) serious dysfunction of organs such as heart, liver, and kidney; (5) primary distant metastasis; and (6) pregnant and lactating mothers.

### Treatment regimen

**Figure 1:**
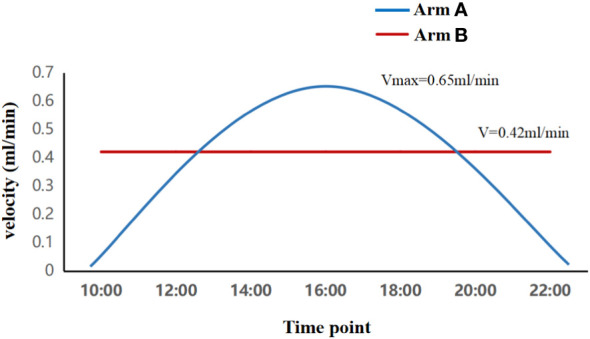
Different administration methods of cisplatin in two groups. Patients in Arm A underwent sinusoidal chrono-modulated infusion, and those in Arm B underwent flat intermittent constant rate infusion.

All patients received 2 cycles of docetaxel, DDP, and fluorouracil (TPF) based IC in 21 day cycles followed by CCRT. The IC with docetaxel and DDP at 75mg/m^2^ administered through bolus infusion on the first day. Further, fluorouracil at 750 mg/m^2^ for 5 days was administered as continuous intravenous pumping. IMRT was administered once a day, five times a week (Monday to Friday) for 6 to 7 weeks. The radiation dose of target areas were set as GTVnx (gross tumor volume of the nasopharynx): 69.96Gy-73.92Gy/33 fraction (fr), 2.12-2.24Gy/1fr; PTVnx (planning target volume of the nasopharynx): 69.96 Gy/33fr, 2.12 Gy/1fr; GTVnd (gross tumor volume of the involved lymph nodes): 69.96 Gy/33fr, 2.12 Gy/1fr; CTV1 (clinical volume 1, high-risk clinical target volume): 60.06 Gy/33 fr, 1.82 Gy/1fr; and CTV2 (low-risk clinical target volume): 50.96 Gy/28 f,1.82 Gy/1fr. Chrono-chemotherapy was carried out during radiotherapy. Patients in Arm A were given 100 mg/m^2^ cisplatin from 10:00 am to 10:00 pm (peaked at 04:00 pm) on Day 1. With sinusoidal administration, the maximum velocity (Vmax) of administration during peak time was 0.65ml/min. Patients in Arm B received conventional intravenous infusion of 100 mg/m^2^ cisplatin from 10:00 am to 10:00 pm on Day 1. The uniform administration velocity was 0.42ml/min. The CCRT was administered in 21-day cycles for 2-3 cycles. The administration modes of the two groups are shown in **Figure 1**.

### Follow-up

All patients were followed up every 3 months during the first 2 years, every 6 months during the third to fifth year, and then annually thereafter. During follow-up, the data was reviewed for blood and biochemistry profiles, magnetic resonance imaging of nasopharynx and neck, fibrous nasopharyngoscopy, chest computed tomography (CT) or chest X-ray, upper abdominal CT or abdominal ultrasound, and emission CT based on the patient’s clinical symptoms. Patients were followed up on an outpatient basis and their survival and long-term toxic side effects were recorded. Patients who did not return for follow-up were contacted by phone to assess their survival and side effects. Adverse reactions were evaluated by Common Terminology Criteria for Adverse Events (CTCAE 3. 0) (15). All patients were followed up until November 30, 2021, or death from any cause. The primary endpoint of the study was progression-free survival (PFS) calculated from the time of enrollment until first recurrence at any site, death from any cause, or patient examination at last follow-up. The secondary endpoints were OS defined as the time from registration to death from any cause, distant metastasis–free survival (DMFS), and local recurrence–free survival (LRFS). DMFS and LRFS were defined as the time from patient admission until first distant metastasis and local recurrence, respectively. Late toxic side effects were defined as those that occurred 6 months after completion of radiotherapy. Side effects were assessed and graded based on the Radiation Therapy Oncology Group/European Organization for Research and Treatment of Cancer morbidity-scoring schema.

### Statistical analysis

The Statistical Package for Social Sciences (SPSS) software, version 24.0 (SPSS Inc), was used for statistical analyses. The incidence of late toxic side effects and other categorical variables were compared using the χ2 test or Fisher’s exact test as appropriate. Survival curves were plotted using Kaplan–Meier method, and log-rank test was conducted. Hazard ratios (HRs) with 95% confidence intervals (CIs) were calculated using a Cox proportional hazards regression model. Measurement data were expressed as mean ± standard deviation, and t-test was used for comparison between the two groups. Interaction and stratified analyses were conducted based on age, sex, WHO histologic grade, smoking status and cancer stage. Interaction is the situation wherein the association of one risk factor with a certain outcome variable differs across the strata of another risk factor (8). In this study, treatment methods and other potential prognostic factors (age [>47 or ≤47], sex [female or male], cancer stage [III or IVA], WHO histologic grade [II or III],smoking status[without or with]) were entered into the multivariate Cox proportional hazards regression model to test for their main effects, and an interaction term between treatment methods and the potential prognostic factors was then added into the model to test their interaction effect on survival. All statistical tests were two-sided, and a P value <0.05 was deemed statistically significant.

## Results

### Patient characteristics

**Figure 2:**
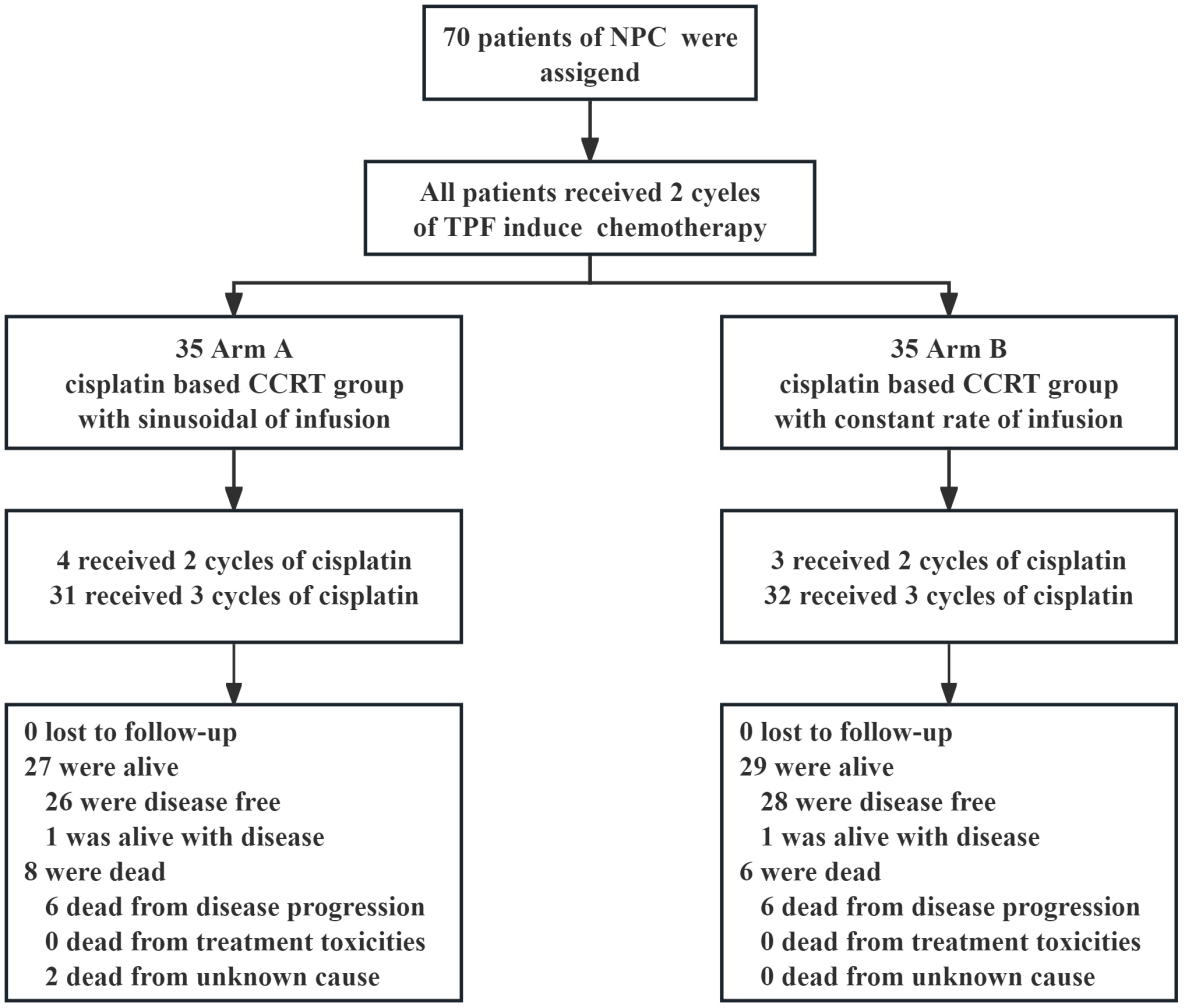
Flowchart of Patients Included and Excluded in This Study.

In the initial and updated analyses, 70 patients were evaluated retrospectively. There were 35 cases each Arm A and Arm B. The patient selection profile is shown in **Figure 2**. The two treatment groups were well matched on baseline demographic and clinical characteristics (**Table 1**). All patients were diagnosed with non-keratinizing differentiated or undifferentiated NPC (WHO type II and III). The median patient age was 47 years (range, 18 to 70 years), and 81.4% of the patients were male; 45 (64.3%) patients had a history of smoking; and 63 (90%) patients had T3 or T4 primary tumors. Most patients had a nodal status of either N1 (25.7%) or N2 (64.3%).

**Table 1:** Baseline characteristics of the study participants.

Characteristic	Arm A (n=35)	Arm B (n=35)
	n (%)	n (%)
Age, median (range), y	46 (18-68)	48 (27-70)
Sex		
Male	29 (82.9)	28 (80)
Female	6 (17.1)	7 (20)
Smoking history		
With	21 (60)	24 (68.6)
Without	14 (40)	11 (31.4)
Tumor classification		
T1	2 (5.7)	0 (0)
T2	2 (5.7)	3 (8.6)
T3	6 (17.1)	5 (14.3)
T4	25 (71.5)	27 (77.1)
Node classification		
N0	0	1 (2.9)
N1	9 (25.7)	9 (25.7)
N2	22 (62.9)	23 (65.7)
N3	4 (11.4)	2 (5.7)
Staging		
III	8 (22.9)	6 (17.1)
IVA	27 (77.1)	29 (82.9)
WHO histologic grade		
II	8 (22.9)	6 (17.1)
III	27 (77.1)	29 (82.9)

WHO, World health organization; n, number of patients

### Efficacy

**Figure 3:**
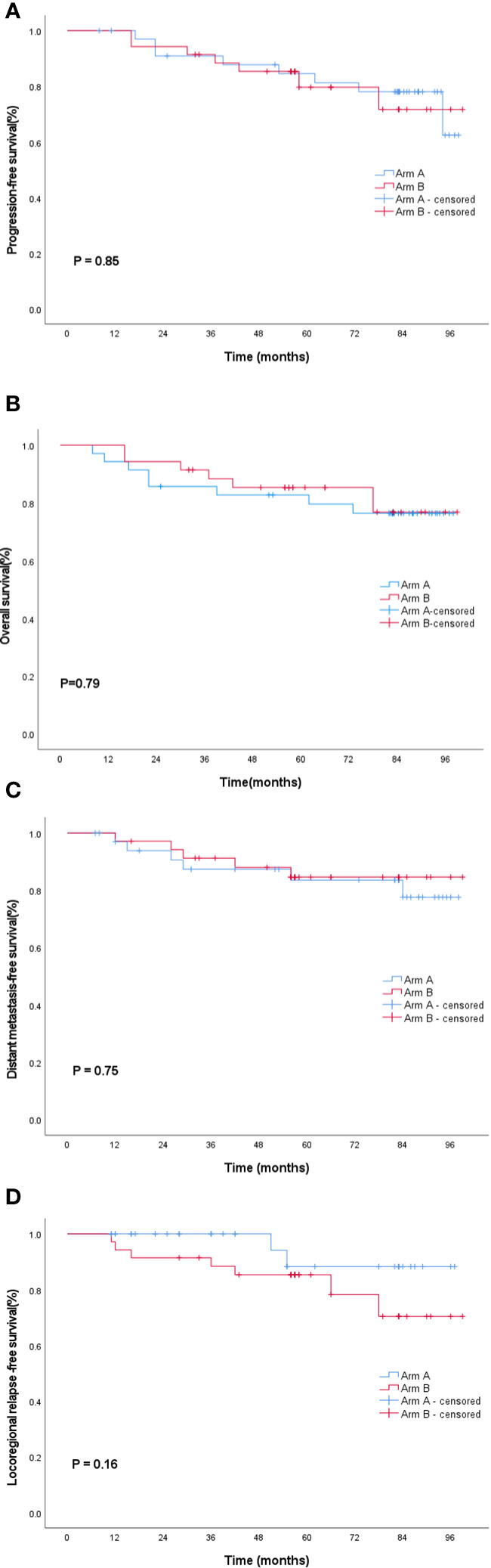
Survival curves of the entire cohort: **(A)** progression-free survival, **(B)** overall survival, **(C)** distant failure-free survival and, and **(D)** local recurrence-free survival. Blue lines indicate Arm A; red lines indicate the Arm B. Arm A, sinusoidal chrono-modulated infusion group; Arm B, constant rate infusion group.

The follow-up period ended on November 30, 2021, with a median follow-up time of 82.8 months. The 5-year PFS rate was 81.3% (95% CI, 76.4-93.6) in Arm A and 79.6% (95% CI, 75.2-94.1) in Arm B (log-rank P = 0.85). No statistically significant difference was found in the 5-year OS between Arm A and Arm B (79.6% vs 85.3%; 95% CI, 72.6-92.5 vs 84.9-98.5; log-rank P = 0.79). Distant metastasis and local recurrence represented a major failure pattern. The 5-year DMFS rate was 83.6% (95% CI, 76.5-95.1) in Arm A and 84.6%(95% CI, 76.5-95.1)in Arm B (P = 0.75). The 5-year LRFS rate was 88.2% (95% CI, 85.1-98.6) in Arm A and 85.3% (95% CI, 74.0-93.9) in Arm B (P = 0.16). As shown in **Figure 3**, the PFS, OS, DMFS, and LRFS curves were plotted using the Kaplan–Meier method, and log-rank test showed no statistically significant differences between Arm A and Arm B.

### Toxicity

Publication of the early results of this trial included details of adverse events during treatment (14). In this long-term analysis, we evaluated the late toxic side effects that occurred after CCRT in Arm A and Arm B. There was no late toxicity of grade 3 to 4 in either group. Dry mouth was the most common late toxic side effect, followed by hearing loss and difficulty in swallowing. Other late toxic side effects included cranial neuropathy, eye damage, neck fibrosis, voice hoarseness, and brain radiation injury (**Table 2**). Although the incidence of 1-2 grade late toxicity in Arm B was higher than that in Arm A, there was no statistically significant difference.

**Table 2:** Late toxic side effects.

Toxicity, n (%)	Arm A				Arm B				χ2	P
	Grade 0		Grade 1-2		Grade 0		Grade 1-2			
**Auditory/hearing**	32	(91.4)	3	(8.6)	30	(85.7)	5	(14.3)	0.57	0.45
**Cranial nerve palsy**	35	(100)	0	(0)	32	(91.4)	3	(8.6)	3.13	0.08
**Dysphagia**	33	(94.3)	2	(5.7)	33	(94.3)	2	(5.7)	0.00	1.00
**Eye damage**	35	(1)	0	(0)	33	(94.3)	2	(5.7)	2.06	0.15
**Neck fibrosis**	35	(1)	0	(0)	34	(97.1)	1	(2.9)	1.01	0.31
**Dry mouth**	28	(0.8)	7	(0.2)	24	(68.6)	11	(31.4)	1.59	0.45
**Voice hoarseness**	35	(1)	0	(0)	32	(91.4)	3	(8.6)	3.13	0.08
**Temporal lobe necrosis**	35	(1)	0	(0)	34	(97.1)	1	(2.9)	1.01	0.31

Differences in adverse events were analyzed using χ2 test;n: number of patients.

### Subgroup analyses

We further performed subgroup analyses for PFS, OS, DMFS, and LRFS in patients stratified by the following covariates: age (≤47 or>47), sex (female or male), disease stage (III or IVA), WHO histologic grade (type II/III), and smoking history (with or without). No interaction was observed between these covariates and the treatment group of PFS (age ≤47 years: HR, 0.78; 95% CI, 0.26-2.22; age >47: HR, 1.28; 95% CI, 0.45-3.64; P = 0.64 for interaction; female sex: HR, 0.49; 95% CI, 0.09-2.56 and male sex: HR, 2.04; 95% CI, 0.39-10.62; P = 0.40 for interaction; stage III disease: HR, 1.81; 95% CI, 0.38-8.74 and stage IVA disease: HR, 0.56; 95% CI, 0.11-2.66; P = 0.46 for interaction; WHO histologic grade type II: HR, 1.14; 95% CI, 0.34-3.84 and grade type III: HR, 0.88; 95% CI, 0.26-2.95; P = 0.83 for interaction; smoking history: HR, 0.86; 95% CI, 0.25-2.96; no smoking history: HR, 1.16; 95% CI, 0.34-4.00; P = 0.81 for interaction; **Table 3**). Similarly, no interaction was observed between these covariates in the two Arms of OS, DMFS, and LRFS. This indicated that the non-inferiority of sinusoidal chrono-modulated infusion group did not differ among specific populations.

**Table 3:** Subgroup analysis **(A)** Progression-free survival.

(A) Progression-free survival								
Characteristics			n/total number of events		HR	95% CI	P for interaction	
			Arm A	Arm B				
Age, y							0.64	
>47			5/14	3/19	1.28	0.45-3.64		
≤47			3/21	4/16	0.78	0.28-2.22		
Sex							0.40	
Female			0/6	2/7	0.49	0.09-2.56		
Male			8/29	5/28	2.04	0.40-10.63		
Stage							0.46	
III			1/8	1/6	1.81	0.38-8.74		
IVA			7/27	6/29	0.55	0.11-2.66		
WHO histologic grade							0.83	
II			2/8	2/6	1.14	0.34-3.84		
III			6/27	5/29	0.88	0.26-2.95		
Smoking history							0.81	
Without			3/14	3/11	1.16	0.34-4.00		
With			5/21	4/24	0.86	0.25-2.96		

(B) Overall survival								
Characteristics			n/total number of events		HR	95% CI		P for interaction
			Arm A	Arm B				
Age, y								0.15
>47			2/21	3/19	2.15	0.72-7.03		
≤47			6/14	3/16	0.50	0.10-2.33		
Sex								0.65
Female			0/6	2/7	0.69	0.22-3.05		
Male			8/29	4/28	1.34	0.17-13.2		
Stage								0.88
III			2/8	1/6	0.88	0.37-4.23		
IVA			6/27	5/29	1.21	0.29-13.31		
WHO histologic grade								0.37
II			2/8	2/6	0.67	0.20-3.01		
III			6/27	4/29	0.54	0.13-3.73		
Smoking history								1
Without			2/14	3/24	1.14	0.36-4.16		
With			6/21	3/11	1.08	0.20-13.19		

(C) Distant metastasis-free survival								
Characteristics			n/total number of events		HR	95% CI		P for interaction
			Arm A	Arm B				
Age, y								0.60
>47			4/14	2/19	1.40	0.40-4.89		
≤47			2/21	3/16	0.72	0.21-2.50		
Sex								0.24
Female			0/6	1/7	0.27	0.03-2.36		
Male			6/29	4/28	3.745	0.42-33.09		
Stage								0.96
III			1/8	1/6	0.96	0.19-4.80		
IVA			5/27	4/29	1.05	0.21-5.24		
WHO histologic grade								0.56
II			1/8	2/6	1.50	0.38-5.97		
III			5/27	3/29	0.67	0.17-2.65		
Smoking history								0.25
Without			3/14	2/11	2.18	0.57-8.31		
With			3/21	3/24	0.46	0.12-1.75		

(D) Locoregional relapse-free survival								
Characteristics			n/total number of events		HR	95% CI		P for interaction
			Arm A	Arm B				
Age, y								0.46
>47			1/14	4/19	1.69	0.43-6.69		
≤47			1/21	3/16	0.59	0.15-2.35		
sSex								0.76
Female			0/6	3/7	1.36	0.19-9.59		
Male			2/29	4/28	0.74	0.10-5.20		
Stage								0.46
III			0/8	1/6	0.45	0.06-3.69		
IVA			2/27	6/29	2.23	0.27-18.28		
WHO histologic grade								0.38
II			1/8	2/6	1.97	0.44-8.90		
III			1/27	5/29	0.51	0.11-2.30		
Smoking history								0.94
Without			0/14	4/11	1.08	0.16-7.17		
With			2/21	3/24	0.93	0.14-6.20		

n, number of patients; HR, hazard ratio; CI, confidence interval; WHO, World health organization. The number of events and patients are shown by study arm. HRs and 95% CIs were calculated using the unadjusted Cox proportional-hazards model; interaction and stratified analyses were conducted according to age, sex, stage, WHO histologic grade, and smoking history.

## Discussion

More than 70% of cases of newly diagnosed NPCs present with locally advanced disease (stage III/IV according to the sixth AJCC staging system) (16). CCRT followed by IC is the standard treatment mode for locally advanced NPC (17–20). Studies have shown that radiotherapy using IMRT can prolong the long-term survival in NPC compared with 2-dimensional radiotherapy (2DRT) (21, 22). The effects of concurrent radio- and chemotherapy drugs complement each other; however, although chemotherapy drugs can improve radiosensitivity, radiotherapy can enhance cytotoxicity. Therefore, the efficacy of concurrent radio- and chemotherapy in locally advanced NPC is better than that of radiotherapy alone (20, 23, 24). The standard chemotherapeutic regimen for NPC is DDP (100 mg/m^2^ in 21-day cycles). Although the efficacy of CCRT has improved, the incidence of adverse reactions has also increased, particularly the acute toxic reaction of DDP (25–27). Therefore, it is important to seek an effective treatment scheme for reducing the toxicity and side effects of cisplatin during CCRT.

The cell rhythm in malignant tumors is significantly different compared to normal cells (28). Disrupted biorhythms or circadian clock genes that are suppressed or mutated can trigger a variety of diseases including malignant tumors (29–33). Similar to most biological functions that are subject to circadian changes (34), pharmacodynamics and pharmacokinetics are influenced by circadian rhythms (35). Pharmacokinetics determines the optimal drug concentration required to produce a balance between efficacy and toxicity (36, 37). Drugs, such as anti-mitotic agents, anti-metabolites, alkylating agents, or inserters, usually achieve an optimal anti-tumor efficacy when used at the time of day when they are best tolerated, but this property is not always used for our own characteristic benefits (38). In contrast, levels of glutathione, an antioxidant molecule involved in drug withdrawal, peak at 04:00 pm. It has been reported that toxicities of certain drugs were decreased when those drugs were administered during the glutathione time of action (32). Therefore, chronotherapy or the pharmacology of clinical chronotherapy study the impact circadian rhythms have on the response to a drug to optimize its action, maximize health benefits, and minimize possible adverse effects on patients (39).

Chrono-chemotherapy is precisely based on the biological rhythm differences of human tumor tissue, normal tissue, and drug metabolism. It involves selecting the time period when chemotherapy drugs have the optimal efficacy on tumor tissue and the lowest toxicity to normal tissue and allows choosing the time of peak drug concentration with the help of a multi-channel programming infusion pump (40). In recent years, chrono-chemotherapy, as a part of the comprehensive treatment of cancer, has been applied in clinical practice locally and internationally and has shown good therapeutic effect in different tumor types. Such as a study showed that irinotecan tolerability was better after morning administration in men and afternoon administration women with metastatic colorectal cancer (41). Studies have shown that in colorectal cancer, the optimal time for oxaliplatin administration is 04:00 pm. In addition, the combination of oxaliplatin, 5-fluorouracil, and calcium folinate (ChronoFLO4) has a survival advantage fewer adverse side effects to the digestive tract in men with colorectal cancer and has (42–46). In non-small cell lung cancer, chronotherapy with DDP decreases hematological and gastrointestinal adverse effects (47). In studies of renal cell carcinoma, administration in accordance with circadian rhythm regulation (68% of the daily dose administered in the evening) induced a durable tumor response with less drug toxicity (47). Morning administration of temozolomide in glioblastoma increased OS in O6-Methylguanine-DNA-methyltransferase (MGMT) methylated patients, which was consistent with the peak expression of the clock gene BMAL1. Patients with glioblastoma may benefit from chrono-chemotherapy (48). Studies have shown that in the treatment of ovarian cancer, DDP administered from 04:00 pm to 08:00 pm and doxorubicin administered at 06:00 am showed minimal drug toxicity and side effects and high tumor response (32, 38). In NPC, induced chrono-chemotherapy combined with radiotherapy enhances tolerance during treatment and reduces treatment-related side effects including thrombocytopenia, leukopenia, nausea, and vomiting (10, 11). The above studies illustrate the superiority of chronotherapy.

The purpose of this study was to evaluate the difference between sinusoidal administration and conventional uniform administration of DDP for an optimal duration in CCRT after IC. In this long-term follow-up analysis, the median follow-up duration was 82.8 months. The 5-year survival results were consistent with those at 2 years. Patients from Arm A achieved comparable 5-year PFS, OS, DMFS, and LRFS rates as those in Arm B. The two groups in this study were well balanced in terms of patient characteristics, tumor factors, and treatment parameters. In the subgroup analysis, no interaction was observed between these covariates and the groups, indicating that the non-inferiority of Arm A did not differ among specific populations. This also suggests that there is no survival benefit to patients with different infusion rates of chrono-chemotherapy during long-term follow-up.

In terms of long-term side effects, there were no grade 3-4 side effects in both groups. In Arm A and Arm B, the long-term toxic side effects were grade 1-2, including dry mouth, dysphagia, and hearing loss. Patients in group B also developed grade 1-2 cranial neuropathy, eye damage, neck fibrosis, voice hoarseness, and radiation brain radiation injury. Although there was no statistically significant difference between the two groups in the long-term toxic and side effects, it can be seen that the number of cases of long-term toxic and side effects in Arm B was higher than that in Arm A. This may be because the number of T4 stage cases in Arm B was slightly more compared to Arm A. The relatively large radiotherapy target area of T4 stage patients was possibly related to the differences in tolerance among patients. In the previous reports of this study, there was no statistically significant difference in acute toxic and side effects between the two groups during CCRT. Although studies have reported that sine administration of DDP and fluorouracil in TPF regimen induction chemotherapy has no survival advantage, it can reduce the incidence of stomatitis (11). The difference between our results and the above results is considered to be due to the different treatment stages. The effect of stomatitis caused by radiotherapy was more prominent than that caused by chemotherapy during CCRT. In addition, fluorouracil was the main factor leading to oral mucositis during IC, and there was no fluorouracil drug involved in the CCRT. Therefore, compared to the constant infusion rate of chemoradiotherapy, the sinusoidal form of administration during the CCRT did not improve the efficacy or reduce the adverse reactions related to chemoradiotherapy. However, we could not compare the sine administration and constant infusion rate of IC and CCRT, which will be considered in our future study. Besides, further research should be conducted to determine the optimal chronotherapy schedule for NPC.

T lymphocytes are a type of lymphocyte that plays a central role in cell-mediated immunity. Chemotherapy may have an inhibitory effect on immune cells, leading to immune dysfunction (49).In the previous report of this study, the CD3+ value of the sine group was higher than that of the constant infusion rate group after treatment, and the difference was statistically significant. It indicates that sine rate administration could improve the T-cell immune function of patients compared with constant infusion (14). Unfortunately, in this long-term follow-up, due to the limited inspection conditions, we could not detect the immune lymphocyte subsets in some patients visiting their hometown hospital for review and telephone follow-up. Therefore, we could not further analyze the long-term detection results of the immune lymphocyte subsets of these patients. At present, immune checkpoint therapy targeting Programmed death 1 (PD-1) receptor and its ligand PD-L1 has been approved for the treatment of patients with certain types of malignancies (50).Immunotherapy drugs have shown good anti-tumor activity in patients with advanced NPC or with recurrence or metastasis after failure of standard therapy (51). In recent findings, the antitumor efficacy of PD-1/PD-L1 inhibitor varies according to its administration time. This suggests that the selection of the most appropriate dosing time for PD-1/PD-L1 inhibitors is helpful to improve the efficacy of immunotherapy (52). Whether chronotherapy can be used in immunotherapy is also a question worth exploring.

This study was retrospective with a limited number of patients enrolled and without a comprehensive follow-up. In future, more rigorous multicenter prospective randomized studies with a large sample size should be designed to confirm the research conclusions.

## Conclusions

In this retrospective analysis, long-term analysis confirmed that in the concurrent chemoradiotherapy, cisplatin administration with sinusoidal chronomodulated infusion group was not superior to constant rate of infusion in terms of Long-term toxicity and prognosis.

## Data availability statement

The raw data supporting the conclusions of this article will be made available by the authors, without undue reservation.

## Ethics statement

The studies involving humans were approved by Ethics Committee of Affiliated Cancer Hospital of Guizhou Medical University. The studies were conducted in accordance with the local legislation and institutional requirements. Written informed consent for participation was not required from the participants or the participants’ legal guardians/next of kin in accordance with the national legislation and institutional requirements.

## Author contributions

LL: Conceptualization, Data curation, Formal analysis, Investigation, Methodology, Writing – original draft, Writing – review & editing. XYL: Data curation, Formal analysis, Writing – original draft. WW: Validation, Writing – review & editing. YL: Validation, Writing – review & editing. JL: Validation, Writing – review & editing. XLL: Data curation, Investigation, Writing – review & editing. XC: Data curation, Investigation, Writing – review & editing. XG: Data curation, Investigation, Writing – review & editing. CZ: Data curation, Software, Writing – original draft. QH: Data curation, Software, Writing – original draft. ZL: Data curation, Software, Writing – original draft. KS: Data curation, Investigation, Methodology, Writing – original draft. YC: Data curation, Investigation, Methodology, Writing – original draft. XX: Data curation, Investigation, Writing – original draft. FJ: Supervision, Validation, Writing – review & editing.
